# Pectin De-methylesterification and AGP Increase Promote Cell Wall Remodeling and Are Required During Somatic Embryogenesis of *Quercus suber*

**DOI:** 10.3389/fpls.2018.01915

**Published:** 2019-01-08

**Authors:** Yolanda Pérez-Pérez, Elena Carneros, Eduardo Berenguer, María-Teresa Solís, Ivett Bárány, Beatriz Pintos, Aránzazu Gómez-Garay, María C. Risueño, Pilar S. Testillano

**Affiliations:** ^1^Pollen Biotechnology of Crop Plants Group, Biological Research Center – Spanish National Research Council, Madrid, Spain; ^2^Department of Genetics, Microbiology and Physiology, Complutense University of Madrid, Madrid, Spain

**Keywords:** somatic embryogenesis, cell wall remodeling, pectin, methylesterification, pectin methylesterase, AGPs

## Abstract

Somatic embryogenesis is a reliable system for *in vitro* plant regeneration, with biotechnological applications in trees, but the regulating mechanisms are largely unknown. Changes in cell wall mechanics controlled by methylesterification of pectins, mediated by pectin methylesterases (PMEs) and pectin methyl esterase inhibitors (PMEIs) underlie many developmental processes. Arabinogalactan proteins (AGPs) are highly glycosylated proteins located at the surface of plasma membranes, in cell walls, and in extracellular secretions, with key roles in a range of different processes. In this study, we have investigated changes in two cell wall components, pectins and AGPs, during somatic embryogenesis in *Quercus suber*, a forest tree of high economic and ecologic value. At early embryogenesis stages, cells of proembryogenic masses showed high levels of esterified pectins and expression of *QsPME* and *QsPMEI* genes encoding a PME and a putative PMEI, respectively. At advanced stages, differentiating cells of heart, torpedo and cotyledonary embryos exhibited walls rich in de-esterified pectins, while *QsPME* gene expression and PME activity progressively increased. AGPs were detected in cell walls of proembryogenic masses and somatic embryos. *QsLys-rich-AGP18, QsLys-rich-AGP17*, and *QsAGP16L1* gene expression increased with embryogenesis progression, as did the level of total AGPs, detected by dot blot with β-glucosyl Yariv reagent. Immuno dot blot, immunofluorescence assays and confocal analysis using monoclonal antibodies to high- (JIM7, LM20) and low- (JIM5, LM19) methylesterified pectins, and to certain AGP epitopes (LM6, LM2) showed changes in the amount and distribution pattern of esterified/de-esterified pectins and AGP epitopes, that were associated with proliferation and differentiation and correlated with expression of the *PME* and *AGP* genes analyzed. Pharmacological treatments with catechin, an inhibitor of PME activity, and Yariv reagent, which blocks AGPs, impaired the progression of embryogenesis, with pectin de-esterification and an increase in AGP levels being necessary for embryo development. Findings indicate a role for pectins and AGPs during somatic embryogenesis of cork oak, promoting the cell wall remodeling during the process. They also provide new insights into the regulating mechanisms of somatic embryogenesis in woody species, for which information is still scarce, opening up new possibilities to improve *in vitro* embryo production in tree breeding.

## Introduction

*Quercus suber* L. (cork oak) is a forest species of high economic and ecologic value in the Mediterranean area. Cork oak supports a sizeable industry that uses cork as a raw natural material for production of wine bottle-stoppers or thermal and acoustic insulation products, among many other products with applications in construction and space industries. Moreover, cork harvest does not harm the tree, which makes its collection a sustainable and environmentally friendly practice for the forest.

Somatic embryogenesis is considered a feasible system for *in vitro* plant regeneration and is very useful in various biotechnological applications in plant breeding, propagation and conservation strategies (Germana and Lambardi, 2016; Loyola-Vargas and Ochoa-Alejo, 2018; Mohan Jain and Gupta, 2018). This technology is especially useful for woody plants that have a long life cycle and limitations in terms of their propagation by conventional methods, as well as difficulties in terms of seed conservation and vegetative reproduction (Germana, 2009; Guan et al., 2016). Somatic embryogenesis has great potential for large-scale propagation, germplasm conservation and cryopreservation of elite genotypes of trees (Von Arnold et al., 2002; Feher, 2015; Guan et al., 2016; Mohan Jain and Gupta, 2018). In *Q. suber*, somatic embryogenesis has been developed, and protocols for induction and proliferation in several somatic embryogenesis systems have been established (Bueno et al., 1992; Manzanera et al., 1993; Hernandez et al., 2003; Testillano et al., 2018). Despite the clear potential of somatic embryogenesis in woody species, efficiency is very low and variable in many trees, since the mechanisms that control the cellular processes underlying somatic embryogenesis are not yet fully understood.

In addition to other cellular processes, modifications in cell wall components have been reported as being crucial for initiating cell responses in relation to cell fate and development. Plant cell walls are dynamic and complex structures that play important roles in the regulation of plant growth, development, intercellular communication and defense, as well as in the determination of cell shape and fate (Somerville et al., 2004). Growth and differentiation requires remodeling of wall polysaccharide networks during development and in response to external signals (Barnes and Anderson, 2018). Several reports have provided increasing evidence of the crucial role of cell wall components such as pectins and arabinogalactan proteins (AGPs) during somatic and zygotic embryogenesis in plants (van Hengel et al., 2002; Samaj et al., 2005; El-Tantawy et al., 2013; Rodriguez-Sanz et al., 2014).

Pectins are major components of the primary plant cell walls that are secreted into the cell wall in a highly methylesterified form and can be de-esterified *in muro* by pectin methylesterases (PMEs) (Pelloux et al., 2007). The de-methylesterified homogalacturonan domain of pectins can either form Ca^2+^ bonds or become a target for pectin-degrading enzymes, such as polygalacturonases, affecting the texture and rigidity of the cell wall (Pelloux et al., 2007). Changes in the methylesterification status of pectins, controlled by PMEs and pectin methylesterase inhibitors (PMEIs) have been related to the cell wall remodeling that occurs during diverse plant developmental processes (Willats et al., 2001a,b; Baluska et al., 2002, 2005). Recent reports have indicated that changes in cell wall mechanics controlled by the esterification/de-esterification status of pectins underlie organogenesis initiation, early embryo growth and embryogenesis progression (Levesque-Tremblay et al., 2015a,b). Nevertheless, the functional significance of pectin-related cell wall remodeling in different cell types and processes remains unclear.

In addition to polysaccharides, most plant cell walls contain variable amounts of structural proteins such as extensins and AGPs. AGPs are a complex and large superfamily of highly glycosylated hydroxyproline-rich proteins that are present in cell walls, on the surface of plasma membranes and extracellular secretions; they play key roles in several plant developmental processes (Seifert and Roberts, 2007), specifically, they have been implicated in different aspects of sexual reproduction and embryogenesis (Chapman et al., 2000; Zhong et al., 2011; Losada and Herrero, 2012, 2014; Losada et al., 2014; Pereira et al., 2014, 2016; Costa et al., 2015; Lopes et al., 2016). AGPs are structurally very heterogeneous due to their various protein backbones, as well as the extent and degree of arabinogalactan polysaccharide addition. The carbohydrate part is usually in the form of type II arabinogalactan (AG) chains that are *O*-glycosidically linked to Hyp residues on the protein backbone (Ellis et al., 2010). According to their protein backbone composition, AGPs are classified into classical AGPs, AG peptides (with short peptide backbones of 10–15 amino acids), lysine (Lys)-rich AGPs, and chimeric AGPs (Seifert and Roberts, 2007; Yang et al., 2007; Showalter and Basu, 2016). Most AGPs are tethered to the plasma membrane by a glycosylphosphatidylinositol (GPI) making it possible for them to be positioned on the plasma membrane surface outside the cell, with regions of these macromolecules being localized in the cell wall (Seifert and Roberts, 2007; Ellis et al., 2010). The addition of exogenous AGPs to culture medium has been reported to promote somatic embryogenesis in several plant species, including trees (Thompson and Knox, 1998; Yuan et al., 2012; Smertenko and Bozhkov, 2014). In addition, the presence of AGPs secreted from cells into the culture medium has been reported to be a stimulating factor for embryo development in maize microspore and zygote cultures (Borderies et al., 2004; Testillano et al., 2010), as well as in carrot embryogenic suspension cultures (van Hengel et al., 2001). However, the precise role of endogenous AGPs in the regulation of somatic embryogenesis remains poorly understood.

In this study, we have investigated changes in pectin esterification and AGPs during somatic embryogenesis in *Quercus suber*, as well as their possible involvement in this process. The study has been performed by using several complementary approaches which were applied at specific developmental stages of the process: expression analyses of genes encoding a *PME*, a putative pectin methylesterase inhibitor (*PMEI*) and three different AGPs; PME activity assays; immuno dot blot and immunofluorescence assays with monoclonal antibodies to AGPs, high- and low-methylesterified pectins; and analyses of total AGPs levels using the β-glucosyl Yariv reagent which binds AGPs. Functional analyses were also carried out with pharmacological treatments using catechin—an inhibitor of PME activity—and Yariv reagents—to block AGPs. The findings indicate the involvement of pectins and AGPs in somatic embryogenesis of cork oak, may be associated with the remodeling of the cell wall.

## Materials and Methods

### Somatic Embryogenesis Cultures

Somatic embryogenesis was induced in cork oak from immature zygotic embryos (Bueno et al., 1992), following the updated protocol recently described (Testillano et al., 2018). Immature pollinated acorns were collected from trees (late August and September) in El Pardo forest, Madrid, Spain. Briefly, immature acorns at the responsive stage of early cotyledonary embryos were cultivated in induction medium, containing 2,4D (Sigma-Aldrich, Saint Louis, MO, United States), at 25°C with 16/8 h light/darkness. After 1 month, they were transferred to a regulator-free medium (without 2,4D), where proembryogenic masses and somatic embryos developed. By monthly renewal of the same medium, somatic embryogenesis cultures continued their development and multiplied, producing new proembryogenic masses and somatic embryos during months (Testillano et al., 2018).

### *In vitro* Treatments

Clusters of proembryogenic masses with some emerging small embryos were selected and transferred to plates with culture media with the same composition but containing small molecules with reported activity as inhibitors of PMEs (catechins) and blocking agents of AGPs (Yariv reagents, Biosupplies, VIC, Australia). To inhibit PME activity, culture medium was supplemented with 1.5 mg/ml catechin PP60 (Sigma-Aldrich, Saint Louis, MO, United States) (Lewis et al., 2008). To block AGPs, Yariv reagents (Yariv et al., 1962, 1967) were added to culture medium at 300 μg/ml. β-Glucosyl-Yariv phenylglucoside reagent (β-Gluc-Yariv) was used to block AGPs, while β-mannosyl-Yariv phenylglucoside reagent (β-Man-Yariv), which did not interact with AGPs (Tang et al., 2006; Paulsen et al., 2014) was used as control. Treatments were performed during 30 days, with three replicates. Untreated and treated cultures were observed and individual plates were photographed at the time of treatment initiation (day 0) and after 30 days, to monitor culture evolution.

The effect of the treatments on somatic embryogenesis was assessed by quantification of the number of differentiated embryos formed after 30 days from isolated proembryogenic masses (with similar morphology and size), in untreated and treated cultures. Data were expressed as mean values of number of embryos per proembryogenic mass. Differences among treated and untreated cultures were tested by ANOVA and Tukey’s tests at *p* < 0.05.

### Processing of Samples for Microscopy Analysis

Samples from somatic embryogenesis cultures were extracted from Petri dishes and processed for cytochemical and immunofluorescence assays, followed by microscopic analyses, as previously described (Solís et al., 2016). Different developmental stages of the process were studied: clusters of proembryogenic masses, developing somatic embryos at various developmental stages (heart, torpedo and early cotyledonary embryos), and mature embryos. Briefly, samples were fixed in 4% paraformaldehyde in phosphate buffered saline (PBS) at 4°C, overnight. After some washes in PBS, they were dehydrated in an acetone series and embedded in Technovit 8100 acrylic resin (Kulzer, Vehrheim, Germany), at 4°C. Semithin sections were obtained with an ultramicrotome and placed on slides for further processing. Some of them were stained with toluidine blue and observed under bright field microscopy, for general structure analysis. Other sections were placed on multiwell slides covered with the adherent substance APTES (aminopropyl-triethoxi-silane) (Sigma-Aldrich, Saint Louis, MO, United States), and kept at 4°C until use for immunofluorescence.

### Immunofluorescence Assays and Confocal Microscopy Analysis

Immunofluorescence assays were performed on Technovit 8100 sections following the protocol described by us (El-Tantawy et al., 2013; Solís et al., 2016). Several rat monoclonal antibodies to pectins with high (JIM7, LM20) and low (JIM5, LM19) level of methylesterification, and to various AGP epitopes (LM2, LM6) (PlantProbes, Leeds, United Kingdom) were used (Supplementary Table 1). Sections were treated as follows: PBS for 1 min; 5% bovine serum albumin (BSA) for 5 min; incubation with the primary antibody to pectins or AGPs for 1 h; washing in PBS, three times, 1 min each; incubation with the secondary antibody (anti-rat IgG-conjugated to Alexa 488, Thermo Fisher Scientific, Rockford, IL, United States) diluted 1/25 in PBS for 45 min, in darkness; washing in PBS, three times, 1 min each; staining with DAPI (1 mg/ml) for DNA; washing in PBS and distilled water. After that, sections were mounted in Mowiol and observed in a confocal microscope (Leica SP5, Leica Microsystems, Wetzlar, Germany). In order to make appropriate comparison among fluorescence signals, the same settings for sample excitation and capture of emission were kept in the confocal microscope for each antibody in all samples. Controls were performed by omitting the primary antibody.

### Quantitative Real-Time PCR (RT-qPCR)

**FIGURE 1:**
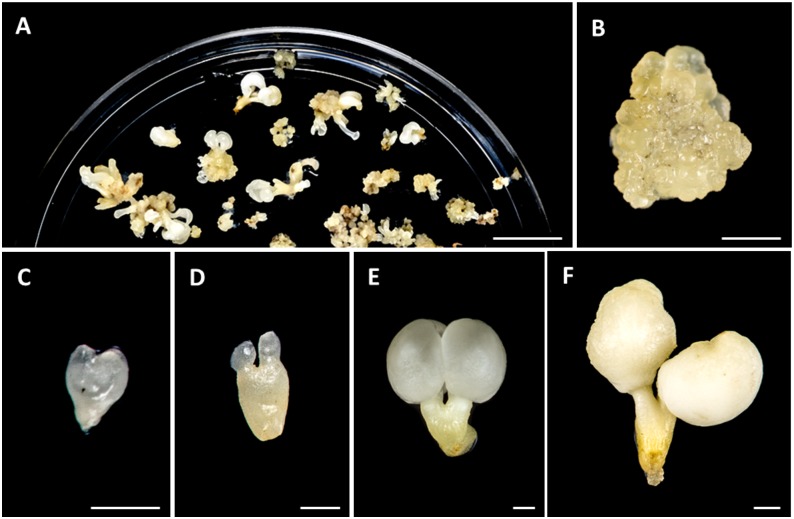
Main stages of somatic embryogenesis of *Quercus suber*. **(A)** Panoramic view of a culture plate showing different structures corresponding to various developmental stages. **(B)** Cluster of proembryogenic masses. **(C)** Heart-shaped embryo. **(D)** Torpedo embryo. **(E)** Cotyledonary embryo. **(F)** Mature cotyledonary embryo. Bars in **(A)**: 1 cm, in **(B–F)**: 1 mm.

Samples from somatic embryogenesis cultures were used for expression analysis experiments at different stages of somatic embryo development (Figure 1): (1) proembryogenic masses; (2) heart, torpedo and early cotyledonary somatic embryos; and (3) mature somatic embryos. Sequences of genes of a *PECTIN METHYL ESTERASE* (*QsPME*, accession number QS073834.0), a putative *PECTIN METHYL ESTERASE INHIBITOR* (*QsPMEI*, accession number QS125571.0) and three *AGPs*, two *LYSINE-RICH AGPs* (*QsLys-richAGP18*, accession number QS117616.0, and *QsLys-richAGP17*, accession number QS005141.0), and an *AG peptide* (*QsAGP16L1*, accession number QS001499.0, (Costa et al., 2015), were selected from the CorkOakDatabase^1^. Gene specific primers were designed with the Primer 3 software (Rozen and Skaletsky, 2000) with default parameters and amendments according to the following criteria: melting temperature around 70°C and product size between 80 and 170 bp (Supplementary Table 2). Total RNA from samples was purified with the NucleoSpin^®^ RNA Plant (Macherey-Nagel, Düsen, Germany) according to the manufacturer’s instructions. RAP buffer with 1% β-mercaptoethanol was used. Contaminating DNA was removed from the total RNA samples using the above-mentioned kit, according to the supplier’s protocol. A 300 ng aliquot of total RNA was used for the reverse transcription reaction using the Superscript^TM^ II Reverse Transcriptase (Invitrogen Life Technologies, Carlsbad, CA, United States) according to the manufacturer’s instructions. RT-qPCR was performed using the FastStart Essential DNA Green Master (Roche Diagnostics, Indianapolis, IN, United States) on the iQ5 Real-Time PCR Detection System (Bio-Rad, Hercules, CA, United States). Thermocycle settings were as follows: initial denaturation of 30 s at 95°C, followed by 40 cycles, each consisting of 5 s at 95°C, 30 s at 58°C. After each run, a dissociation curve was acquired to check for amplification specificity by heating the samples from 58 to 95°C. Serial dilutions of cDNA were used to determine the efficiency curve of each primer pair. As internal reference gene *ACTIN* (*QsACTIN)* was used. A minimum of three biological and three technical replicates were analyzed. Samples of each stage were randomly extracted from at least eight different somatic embryogenesis cultures. Data was analyzed with the Bio-Rad CFX Manager 3.1 (3.1.1517.0823) (Bio-Rad, Hercules, CA, United States), using the Livak calculation method (Livak and Schmittgen, 2001). Transcript levels were normalized using *QsACTIN* values. Data were expressed as mean values of relative expression (fold-change values) to proembryogenic masses sample. Differences among stages were tested by one-way ANOVA analysis of variance followed by Tukey’s multiple comparison test at *P* ≤ 0.05.

### PME Activity Assay

Total protein extracts were obtained from 100 mg samples of different developmental stages of somatic embryogenesis. Samples were homogenized in liquid nitrogen using a mortar, in 60 μl of buffer containing 25 mM Tris–HCl, 50 mM *trans*-1,2-diaminocyclohexane-N, N, N’ N’-tetraacetic acid (CDTA), 25 mM dithiothreitol (DTT) and 1.5% (w/v) polyvinylpolypyrrolidone (PVPP). The resulting supernatant protein concentrations were determined according to Bradford (1976) (Quick-Start Bradford Dye Reagent, Bio-Rad Hercules, CA, United States) using BSA as standard and all samples were adjusted to a concentration of 1 mg/ml. PME activity was determined by a coupled enzymatic assay as described (Levesque-Tremblay et al., 2015a), with 0.4 mM NAD (Sigma-Aldrich, Hercules, CA, United States, Cat. N8410), 0.2 U of alcohol oxidase (Sigma-Aldrich, Hercules, CA, United States, Cat. A2404), 1 U of formaldehyde dehydrogenase (Sigma-Aldrich, Cat. N. F1879) in 100 mM sodium phosphate buffer, pH 7.5, and 5% (w/v) pectin (Sigma-Aldrich, Hercules, CA, United States, pectin from citrus peel, Cat. P-9135) in H_2_O per reaction, using a 96-well plate. The assays were carried out in duplicate. Accumulation of absorbance was detected at 340 nm in a Multiskan^TM^ Sky Microplate UV/Vis Spectrophotometer (Thermo Fisher Scientific, Rockford, IL, United States) and converted into picomoles of NADH produced per minute per milligram of total protein (pmol/min/mg).

### Immuno Dot Blot Assay

The assay was performed essentially as previously described (El-Tantawy et al., 2013; Solís et al., 2016), with minor modifications. Extracts were obtained from different developmental stages of somatic embryogenesis, frozen and homogenized in liquid nitrogen in 50 ml of buffer solution containing 25 mM Tris–HCl, 50 mM *trans*-1,2-diaminocyclohexane-N, N, N’ N’-tetraacetic acid (CDTA), 25 mM DTT and 1.5% (w/v) PVPP. The resulting supernatant protein concentrations were determined according to Bradford (1976) (Bio-Rad Protein Assay reagent) using BSA as standard and all samples were adjusted to a concentration of 1 mg/ml. For immune dot blot assays, 10 μl of adjusted supernatants were applied to a nitrocellulose membrane (Millipore; Bedford, MA, United States) previously activated in methanol for 15 s, in distilled water for 2 min and in TBS for 5 min, and left to dry for 1 h. Then the membrane was incubated for 1 h in blocking buffer (1.5% powdered skimmed milk dissolved in TBS) at room temperature and washed three times for 10 min in TBS. Subsequently, the membrane was incubated overnight at room temperature, with the primary antibody (rat monoclonal JIM5, JIM7, LM19, LM2, LM6, LM20), all diluted 1/100 in TBS, except LM2 which was diluted 1:200. Then, the membrane was washed three times for 10 min in TBS, incubated for 1 h with alkaline phosphatase-conjugated anti-rat antibody diluted 1/1000 in TBS at room temperature and washed again three times for 10 min in TBS. Finally, the epitopes recognized by the antibodies were revealed by treatment with a nitroblue tetrazolium, bromo-chloroindolyl–phosphate (NBT–BCIP) mixture. Controls were performed omitting the primary antibody.

### Yariv Reagent Dot Blot

For Yariv reagent dot blot (Baldwin et al., 1993), 10 μl aliquots of protein extracts from different developmental stages of somatic embryogenesis, all adjusted at the concentration of 1 mg/ml, were applied to a nitrocellulose membrane (Millipore; Bedford, MA, United States) previously activated in methanol for 15 s, in distilled water for 2 min and in PBS for 5 min, and left to dry for 1 h. As positive and negative controls, 10 μL of 2 μg/μL Gum Arabic (Biosupplies, VIC, Australia) containing an AGP mixture, and 10 μL of PBS were applied. Then, the membrane was incubated for 1 h in blocking buffer (1.5% powdered skimmed milk dissolved in PBS, pH 7.4) at room temperature and washed three times for 5 min in PBS, pH 7.4. Subsequently, the membrane was immersed for 15 min in β-Gluc-Yariv or β-Man-Yariv reagents. The concentration of Yariv reagent was 0.15 mg/ml in 1% NaCl. The nitrocellulose membrane was washed in PBS pH 7.4, to decrease background staining before observations were made.

## Results

### Expression Patterns of *QsPME* and *QsPMEI* Genes, and PME Enzymatic Activity During Somatic Embryogenesis

Somatic embryogenesis was induced from immature zygotic embryos, as described in the section “Materials and Methods.” After induction, embryos were produced, either directly from explant or indirectly from proembryogenic masses (PEMs) that were previously formed from explants. Due to the asynchronous development of somatic embryogenesis cultures, different structures corresponding to various developmental stages could be found at the same time point in culture plates (Figure 1A). PEMs appeared in clusters of rounded/nodular masses of cellular aggregates that mostly consisted of proliferating embryogenic cells (Figure 1B). They initially arose from explants, after induction, and their embryogenic cells could either proceeded to from somatic embryos or continued proliferating to form new PEMs (Figure 1A). During *in vitro* culture, somatic embryos were continuously developing, producing globular, heart (Figure 1C), torpedo (Figure 1D) and cotyledonary embryos (Figure 1E), that could be observed together with new PEMs in the culture plates (Figure 1A). PEMs and embryos at different developmental stages suffered recurrent embryogenesis and produced new PEMs and embryos. Spontaneously, some cotyledonary embryos accumulated reserve nutrient substances in cotyledons, which became opaque and ivory-colored, and increased their weight, giving rise to mature somatic embryos (Figure 1F).

**FIGURE 2:**
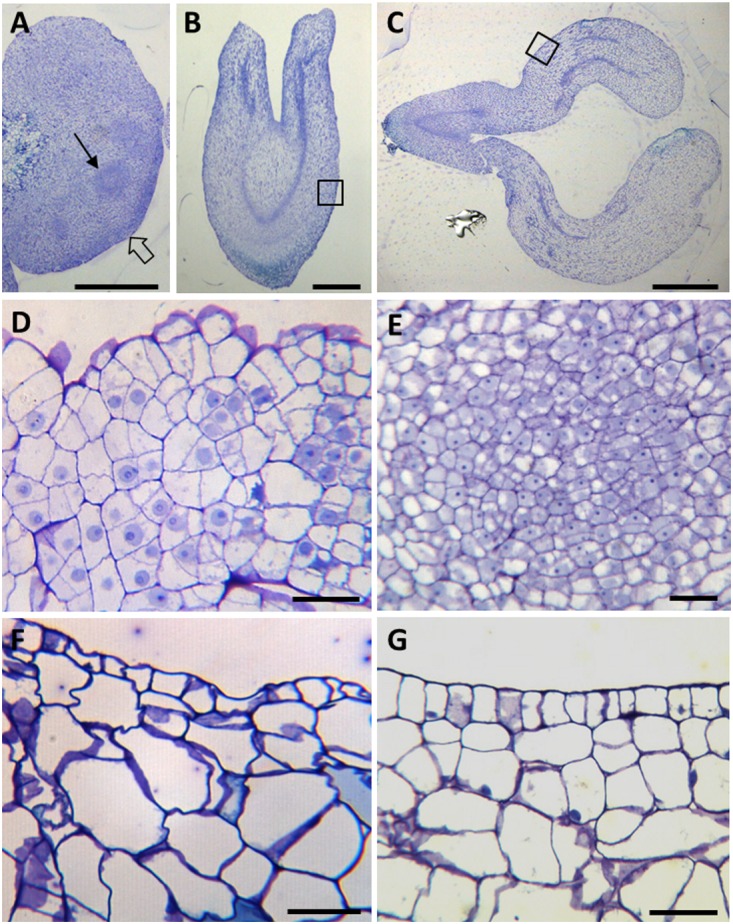
Cellular organization of main stages of somatic embryogenesis. Micrographs of semithin sections stained by Toluidine blue. **(A–C)** Panoramic views of cluster of proembryogenic masses **(A)**, torpedo embryo **(B)** and cotyledonary embryo **(C)**. **(D–G)** Details at higher magnification of representative regions of **(A–C)**, as indicated by arrows and squares. **(D,E)** PEMs showing clusters of embryogenic cells at the periphery **(D)**, as indicated by thin arrow in **(A)**, and inside **(D)**, as indicated by open arrow in **(A)**. **(F)** Torpedo embryo. **(G)** Cotyledonary embryo. Bars in **(A,B)**: 0.5 mm, in **(C)**: 1 mm, in **(D,E)**: 20 μm.

Microscopic analysis revealed that PEMs were formed by aggregates of small embryogenic cells, which appeared in clusters at the periphery of the PEMs (open arrow in Figure 2A, and higher magnification in Figure 2D) or inside PEMs (thin arrow in Figure 2A, and higher magnification in Figure 2E). These cells showed a large central nucleus and a prominent nucleolus, with low vacuolation and a high nucleus-cytoplasm volume ratio (Figures 2D,E), that is the typical structure of proliferating cells. In contrast, as development progressed, somatic embryos at different developmental stages, like heart-shaped, torpedo (Figure 2B) and cotyledonary (Figure 2C) embryos, showed much larger cells, with large vacuoles that occupied most of the cell volume, and small nuclei located at the cell periphery (Figures 2F,G, corresponding to close-up images of embryo regions indicated by squares in Figures 2B,C). At the periphery of embryos, in heart, torpedo and cotyledonary embryos, the differentiating epidermis was observed in transverse sections as a single cell layer of small polygonal cells (Figures 2F,G).

**FIGURE 3:**
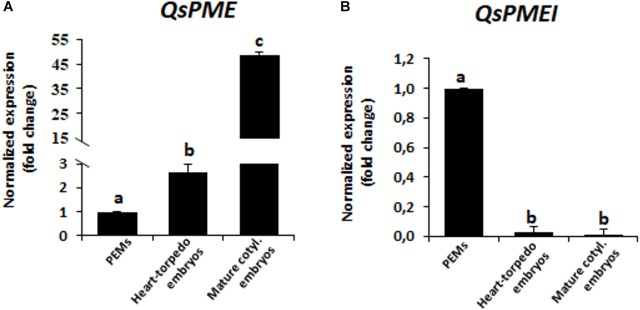
Gene expression patterns of *QsPME*, pectin methyl esterase, and *QsPMEI*, pectin methylesterase inhibitor, genes during somatic embryogenesis by RT-qPCR. Histograms show relative changes of expression at three stages of somatic embryogenesis: proembryogenic masses (PEMs), heart-torpedo embryos and mature cotyledonary embryos. **(A)** Temporal expression pattern of *QsPME* gene. **(B)** Temporal expression pattern of *QsPMEI* gene. Each column represents the mean of at least three biological and three technical replicates. Transcript levels were normalized using *QsACTIN* values. Data were expressed as mean values of relative expression (fold-change values) to proembryogenic masses sample. Bars indicate the standard error of the mean (SEM). Different letters on columns indicate significant differences according to ANOVA and Tukey’s tests at *P* < 0.05.

To study the changes in methylesterification of pectins during somatic embryogenesis, we firstly analyzed the expression of *QsPME* and *QsPMEI*, two genes annotated in the cork oak database as encoding a PME (enzyme that catalyzes the de-methylesterification of pectins) and a PMEI (endogenous proteinaceous inhibitor of the activity of PMEs). The analyses were performed in sequential developmental stages: “proembryogenic masses,” which include proembryogenic masses and early globular embryos arising from them; “heart and torpedo embryos,” stages of embryo differentiation; and “mature cotyledonary embryos.” The results showed that *QsPME* expression was low in PEMs, while it was induced during somatic embryogenesis progression (Figure 3A). *QsPME* expression was threefold higher in heart-torpedo embryos than in PEMs; mature cotyledonary embryos showed the highest expression levels (50-fold higher than PEMs). On the contrary, the *QsPMEI* gene was expressed at early somatic embryogenesis stages (in proembryogenic masses), and it was down-regulated at more advanced developmental stages, in heart-torpedo and mature cotyledonary embryos, which showed almost no expression (Figure 3B).

**FIGURE 4:**
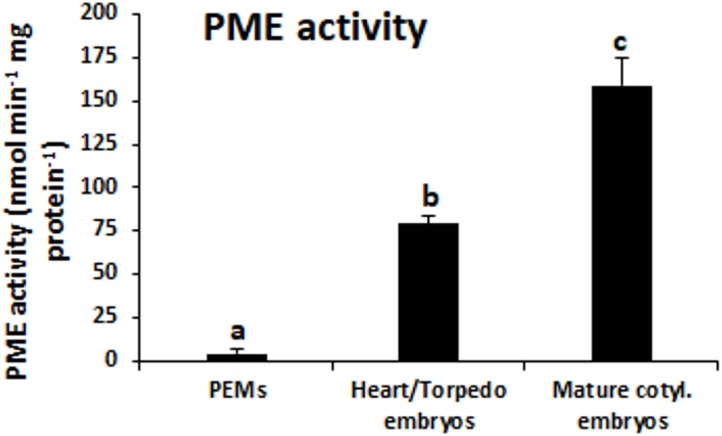
PME activity during somatic embryogenesis. Histogram expresses enzymatic activity levels at three stages of somatic embryogenesis: proembryogenic masses (PEMs), heart-torpedo embryos and mature cotyledonary embryos. Columns represent mean values and bars indicate the standard error of the mean (SEM). Different letters on columns indicate significant differences according to ANOVA and Tukey’s tests at *P* < 0.05.

Pectin methylesterase enzymatic activity was quantified in protein extracts at the same developmental stages. The results showed that PME activity was very low at initial stages, in proembryogenic masses, whereas it greatly increased in developing embryos, at heart and torpedo stages (Figure 4). At more advanced developmental stages, PME activity increased again, with mature cotyledonary embryos exhibiting the highest level of PME activity (Figure 4). This temporal profile of PME activity during somatic embryogenesis correlated well with the gene expression patterns found for *QsPME.*

### Temporal Patterns and Subcellular Localization of Esterified and De-Esterified Pectins During Somatic Embryogenesis

**FIGURE 5:**
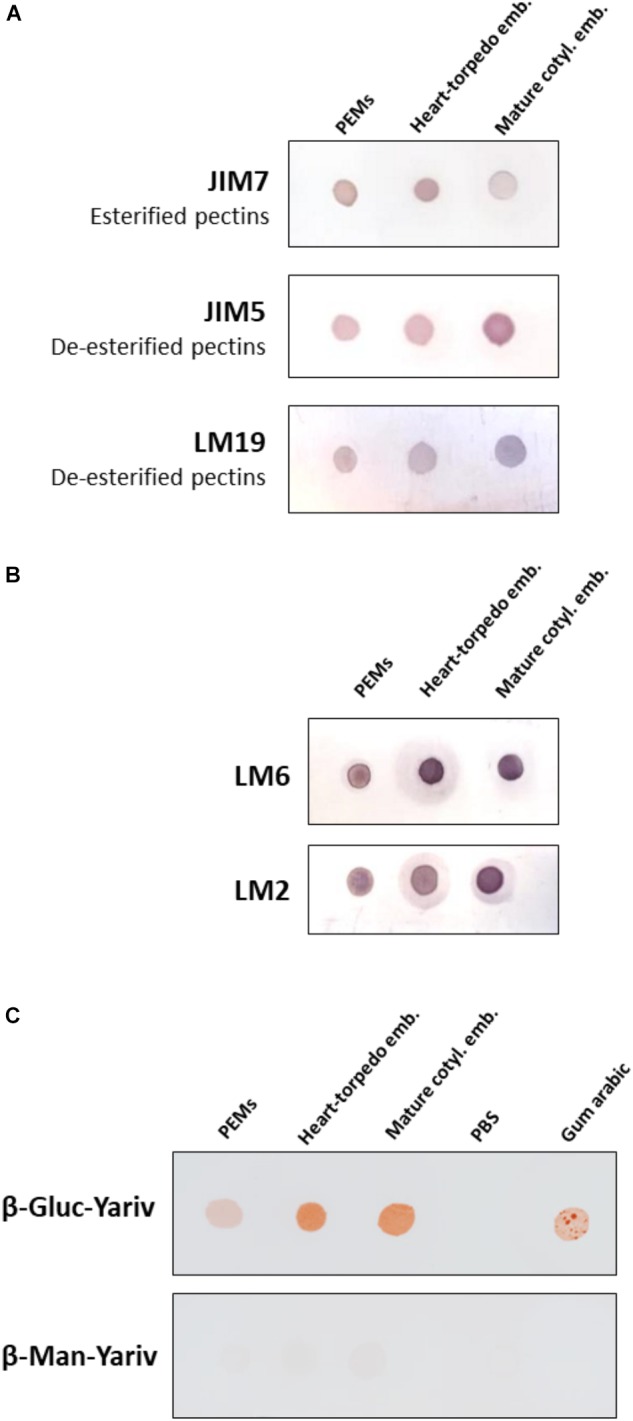
Temporal profiles of esterified/de-esterified pectins and AGPs during somatic embryogenesis. Equal amounts of extracts were dotted for each developmental stage: proembryogenic masses (PEMs), heart-torpedo embryos, and cotyledonary embryos. **(A)** Immuno dot blot assays with JIM7 antibody (esterified pectins), JIM5 antibody (de-esterified pectins), and LM19 antibody (de-esterified pectins). **(B)** Immuno dot blot assays with LM6 and LM2 antibodies for specific AGP epitopes. **(C)** Dot blots stained by β-glucosyl-Yariv reagent, that binds to AGPs, and β-mannosyl-Yariv reagent, which does not bind AGPs; from left to right: PEMs, heart-torpedo embryos, cotyledonary embryos, PBS (negative control) and Gum arabic (positive control). Strip of β-Gluc-Yariv shows increasing color intensity (indicating increase of AGPs) during progression of somatic embryogenesis. Strip control stained with β-Man-Yariv does not provide color signal in any dot.

To analyze the variations in the methylesterification status of pectins during somatic embryogenesis, four monoclonal antibodies were used for dot blot assays with samples from the selected developmental stages: PEMs, heart-torpedo embryos and mature cotyledonary embryos, dotting equal extract volume and protein concentration in all stages. Two different antibodies that specifically recognized highly methylesterified (esterified) pectins (JIM7 and LM20), and two antibodies recognizing low-methylesterified (de-esterified) pectins (JIM5 and LM19) were applied in dot blot experiments. Both antibodies against esterified pectins, JIM7 and LM20, provided similar results; immuno dot blot signal of JIM7 showed the presence of esterified pectins in all stages, with a significant decrease at advanced stages of the process, in mature cotyledonary embryos (Figure 5A). Conversely, immuno dot blots with JIM5 and LM19 antibodies, which label de-esterified pectins, showed an increase in the signal intensity with somatic embryogenesis progression, reaching the highest signal in mature cotyledonary embryos (Figure 5A).

To analyze the patterns of distribution in cell walls of pectins with different levels of methylesterification, immunofluorescence assays were performed using the same set of monoclonal antibodies used in dot blot assays. The experiments were analyzed by confocal microscopy, keeping the settings of excitation and emission capture the same for all samples in each antibody. Under these conditions an accurate comparison of the fluorescence intensity of signals among different developmental stages was possible.

**FIGURE 6:**
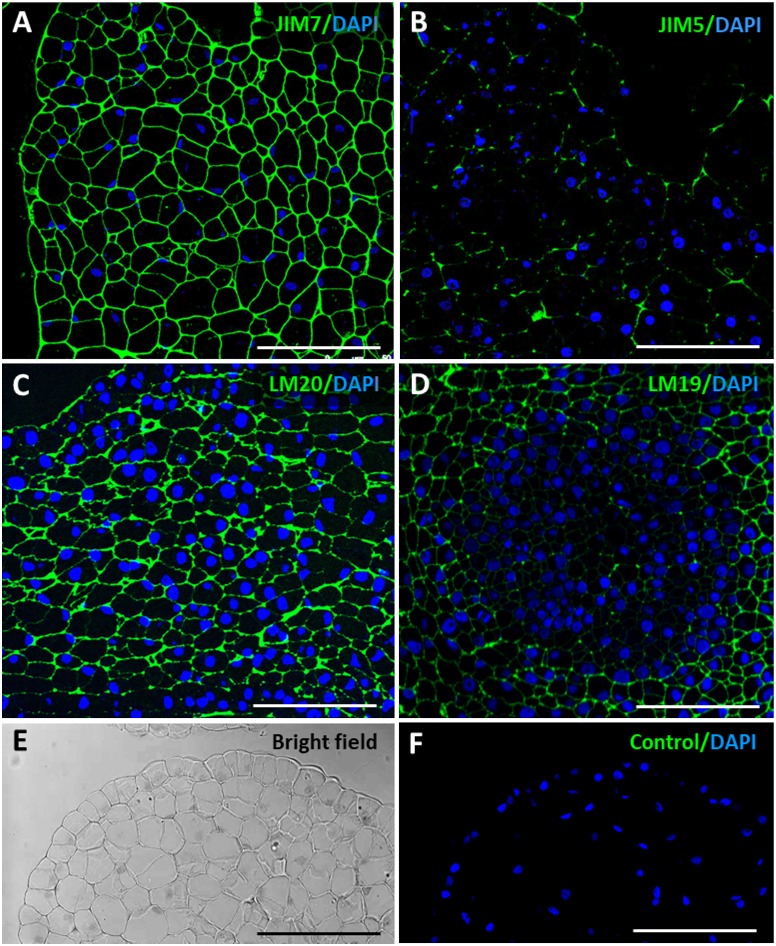
Immunofluorescence of esterified and de-esterified pectins in proembryogenic masses, early stage of somatic embryogenesis. Confocal microscopy images of merged fluorescence signals from pectins (green) and DAPI-stained nuclei (blue). **(A,C)** Immunofluorescence for esterified pectins with JIM7 antibodies **(A)**, and LM20 antibodies **(C)**. **(B,D)** Immunofluorescence for de-esterified pectins with JIM5 antibodies **(B)**, and LM19 antibodies **(D)**. **(A–C)** Clusters of proembryogenic cells of a peripheral region, similar to Figure 2D. **(D)** Cluster of embryogenic cells at the interior, similar to Figure 2E. **(E,F)** Negative control omitting the first antibody, same PEM region visualized under bright field and confocal microscopy **(F)**. Bars represent 20 μm.

The results showed the distribution patterns of esterified and de-esterified pectins in cell walls during somatic embryogenesis initiation and progression. At initial stages, the proembryogenic masses showed intense immunofluorescence signal over all cell walls with antibodies to esterified pectins, JIM7 and LM20 (Figures 6A,C); no other signal or background was observed on any other cellular component. Conversely, labeling for de-esterified pectins, by JIM5 and LM19 antibodies, was very low in the cell walls of proembryogenic masses (Figures 6B,D), in the embryogenic cells localized at the periphery (Figure 6B) and in the clusters of embryogenic cells inside the masses (Figure 6D); they only showed faint signal on the cell corners. These results indicated that cell walls of proembryogenic masses contained a high level of esterified pectins and low levels of de-esterified pectins. Controls in the absence of the primary antibody did not show labeling in any cell compartment at any developmental stage of somatic embryogenesis (Figures 6E,F and Supplementary Figure 1), supporting the specificity of the immunofluorescence results and indicating that samples did not exhibit autofluorescence in the cell walls or any other subcellular structure.

**FIGURE 7:**
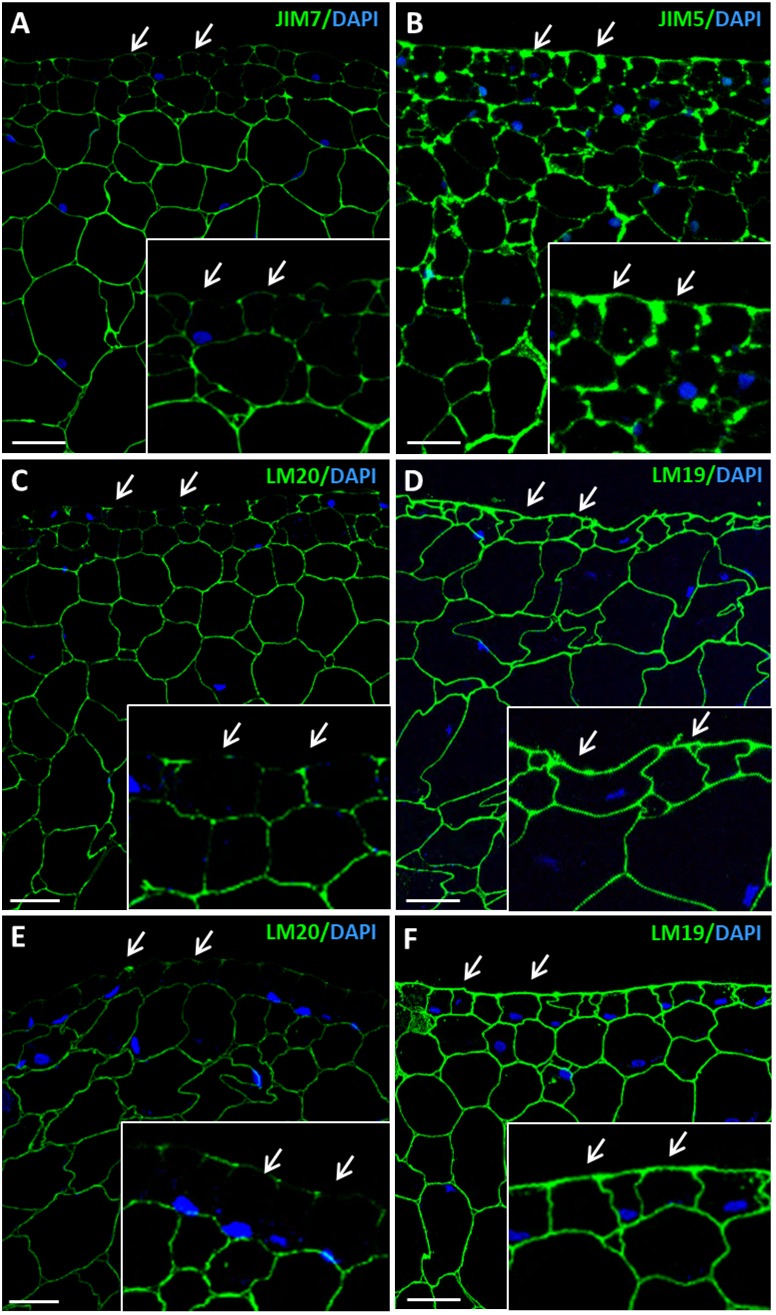
Immunofluorescence of esterified and de-esterified pectins at advanced stages of somatic embryogenesis. Confocal microscopy images of merged fluorescence signals from pectins (green) and DAPI-stained nuclei (blue). **(A,C,E)** Immunofluorescence for esterified pectins with JIM7 antibodies **(A)**, and LM20 antibodies **(C,E)**. **(B,D,F)** Immunofluorescence for de-esterified pectins with JIM5 antibodies **(B)**, and LM19 antibodies **(D,F)**. **(A–D)** Torpedo embryo, region similar to Figure 2F. **(E,F)** Cotyledonary embryo, region similar to Figure 2G. Insets show higher magnification images of epidermis and subjacent tissues. Arrows point to epidermis cells. All pectin antibodies localize in cell walls. Bars represent 20 μm.

As somatic embryogenesis progressed, pectin esterification patterns of labeling changed in developing embryos at different stages, heart, torpedo and cotyledonary embryos. Labeling of esterified pectins (JIM7, LM20 antibodies) was found in the walls of most embryo cells at the heart-torpedo (Figures 7A,C) and cotyledonary stages (Figure 7E), except for the layer of differentiating cells of the epidermis which did not show immunofluorescence signal (Figures 7A,C,E). At the same stages of developing embryos, immunofluorescence signal for de-esterified pectins (JIM5, LM19 antibodies) was very intense in all cell walls of heart, torpedo (Figures 7B,D) and cotyledonary (Figure 7F) embryos, including the cell walls of the epidermis (Figures 7B,D,F) that formed a layer of small polygonal cells all around the embryo body. These immunofluorescence results correlated with those of the immune dot blots indicating that de-methylesterification of pectins increased at advanced stages of somatic embryo development, associated with cell differentiation.

### Gene Expression, Temporal Patterns and Subcellular Localization of AGPs During Somatic Embryogenesis

To study the possible role of AGPs during somatic embryogenesis of cork oak, temporal expression patterns of three AGP genes were analyzed in the three sequential developmental stages previously described, proembryogenic masses, heart-torpedo embryos, and mature embryos. Among the putative AGP sequences found in the cork oak database, we have selected three sequences that encoded two different types of AGPs: two Lys-rich AGPs—*QsLys-rich-AGP17* and *QsLys-rich-AGP18*—and an AG peptide, —*QsAGP16L1*—the latter has been previously identified and characterized in *Q. suber* (Costa et al., 2015). There is very scarce information about AGP sequences in the cork oak database, but there were some reports that related the selected AGPs to embryo formation, which suggested that they could also be expressed in somatic embryogenesis. Expression of *AGP17, AGP18*, and *AGP16* has been reported in siliques, containing embryos (Han et al., 2017); furthermore, *AGP18* has been described as essential in female gametophyte formation (Acosta-García and Vielle-Calzada, 2004).

**FIGURE 8:**
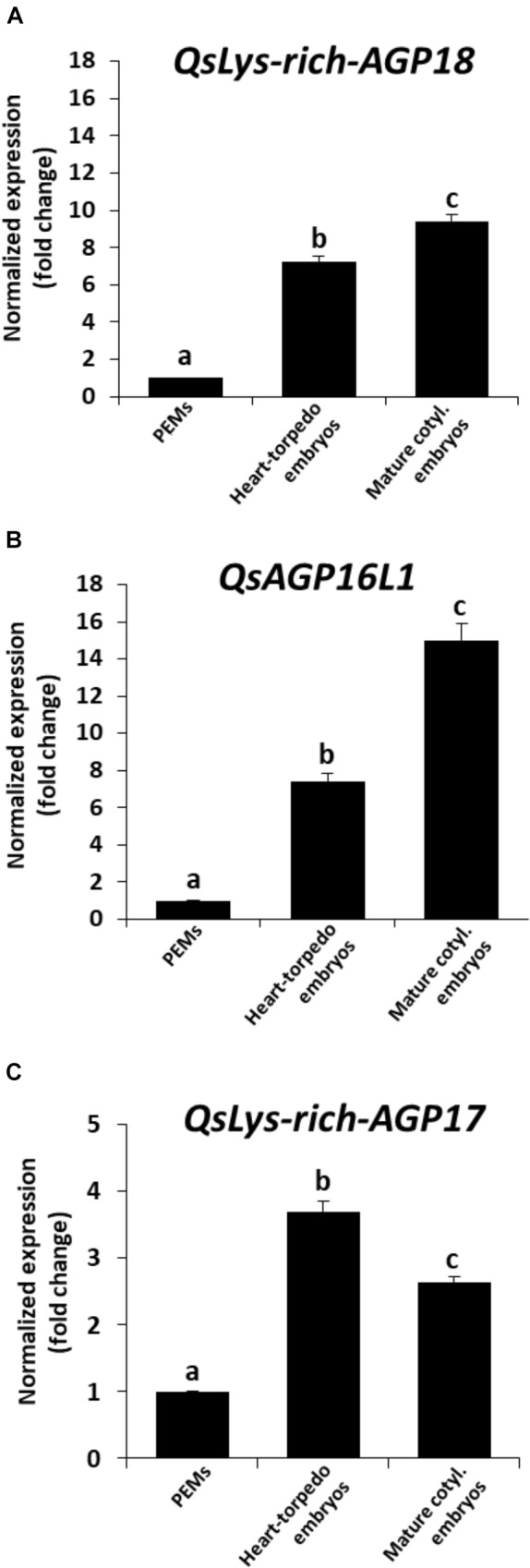
Gene expression patterns of AGP genes during somatic embryogenesis by RT-qPCR. Histograms show relative changes of expression at three stages of somatic embryogenesis: proembryogenic masses (PEMs), heart-torpedo embryos, and mature cotyledonary embryos. **(A)** Temporal expression pattern of *QsLys-rich-AGP18* gene. **(B)** Temporal expression pattern of *QsAGP16L1* gene. **(C)** Temporal expression pattern of *QsLys-rich-AGP17* gene. Each column represents the mean of at least three biological and three technical replicates. Transcript levels were normalized using *QsACTIN* values. Data were expressed as mean values of relative expression (fold-change values) to proembryogenic masses sample. Bars indicate the standard error of the mean (SEM). Different letters on columns indicate significant differences according to ANOVA and Tukey’s tests at *P* < 0.05.

RT-qPCR assays provided temporal profiles of increasing expression with somatic embryogenesis progression for the three AGP genes. They showed expression at initial stages, in proembryogenic masses, and an increase in transcript levels during subsequent developmental stages, in heart and torpedo embryos (Figure 8). *QsLys-rich-AGP18* and *QsAGP16L1* showed an increase in expression of around eightfold in comparison with proembryogenic masses (Figures 8A,B), and *QsLys-rich-AGP17* expression was 4 times higher in heart-torpedo embryos than in proembryogenic masses (Figure 8C). At more advanced stages, in mature embryos, expression increased again for two of the AGP genes analyzed, *QsLys-rich-AGP18* and *QsAGP16L1* (Figures 8A,B); expression of *QsLys-rich-AGP17* slightly decreased in cotyledonary embryos compared with heart-torpedo embryos, maintaining significantly higher expression levels than proembryogenic masses (Figure 8C).

Yariv reagents are synthetic probes that were initially developed as a carbohydrate antigen for the purification of anti-glycoside antibodies and sugar binding proteins (Yariv et al., 1962). Later, these reagents were observed to precipitate AGPs in a wide range of plant extracts (Yariv et al., 1967), being Yariv reagents widely used for the purification of AGPs (Paulsen et al., 2014). To analyze changes in the total content of AGPs at different stages during somatic embryogenesis, β-Gluc-Yariv that specifically interacts with AGPs was used in a dot blot assay. Two different Yariv reagents were used; β-Gluc-Yariv, which is known to interact and aggregate AGPs leading to a brownish precipitate, and β-Man-Yariv, which does not react with AGPs and was used as negative control (Tang et al., 2006; Paulsen et al., 2014). Equal sample amounts of PEMs, heart-torpedo embryos and mature cotyledonary embryos were dotted for the assay, Gum Arabic containing an AGP mixture was used as positive control for the reaction and PBS as negative control. Dot blots with β-Gluc-Yariv showed brown colored dots, indicating the presence of precipitated AGPs, at all somatic embryogenesis stages, as well as in the Gum Arabic dot, while β-Man-Yariv dot blots did not show any color reaction (Figure 5C), supporting the specificity of the results with β-Gluc-Yariv. The intensity of the dot blot signal was lower in proembryogenic masses and increased in advanced stages, in heart-torpedo and cotyledonary embryos (Figure 5C), indicating that the total content of AGPs increased during somatic embryogenesis progression. Negative control with dotted PBS did not show any signal (Figure 5C).

To complement the information obtained with the Yariv dot blot, immuno dot blot assays were performed by using two monoclonal antibodies, LM6 and LM2, which recognized different epitopes of the complex glycosylated structure of the AGPs. LM2 specifically reacts with β-linked-GlcA in AGP glycans (Smallwood et al., 1996). LM6 recognizes α-(1-5)-L-arabinan and therefore has an affinity for AGP arabinans, and can also bind to some chains of the rhamnogalacturonan I domain of pectins (Willats et al., 1998; Verhertbruggen et al., 2009). Both antibodies showed signal at all stages tested and an increase in signal intensity with the progression of somatic embryogenesis (Figure 5B); the proembryogenic mass stage had the lowest labeling intensity, while the cotyledonary embryo stage was the one with the highest, and this was the case for both AGP antibodies (Figure 5B), indicating that these two AGP epitopes increased at advanced stages of somatic embryogenesis. For LM6 epitopes an increase in the immuno dot blot signal intensity was detected in heart-torpedo embryos, while for LM2 epitopes the signal of heart-torpedo embryos was slightly lower (Figure 5B).

**FIGURE 9:**
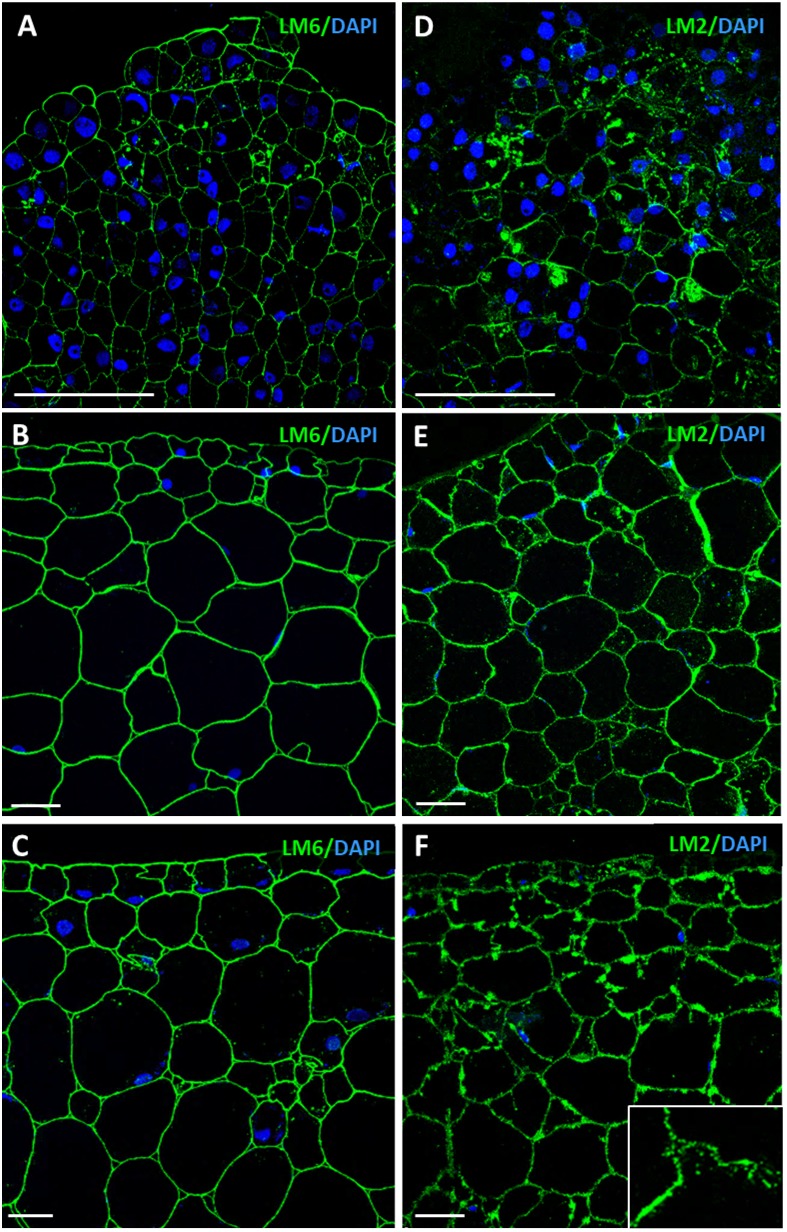
Immunofluorescence of AGP epitopes recognized by LM6 and LM2 antibodies during somatic embryogenesis. Confocal microscopy images of merged fluorescence signals from pectins (green) and DAPI-stained nuclei (blue). **(A–C)** Immunofluorescence for AGP epitopes with LM6 antibodies. **(D–F)** Immunofluorescence for AGP epitopes with LM2 antibodies. **(A,D)** Proembryogenic masses, region similar to Figure 2D. **(B,E)** Torpedo embryo, region similar to Figure 2F. **(C,F)** Cotyledonary embryo, region similar to Figure 2G. Inset show higher magnification image illustrating labeling in cell walls and small cytoplasmic spots. Bars represent 20 μm.

Immunofluorescence assays and confocal analyses were performed to localize AGPs by using LM6 and LM2 monoclonal antibodies. Both antibodies provided specific labeling in cells during initial and advanced developmental stages of somatic embryogenesis, but immunofluorescence intensity was lower at stages earlier than advanced stages, for both antibodies (Figures 9A,D). Labeling intensity with LM6 and LM2 was lower in proembryogenic masses (Figures 9A,D) than in developing somatic embryos where signal intensity increased with embryogenesis progression, in heart, torpedo (Figures 9B,E) and cotyledonary embryos (Figures 9C,F). The localization pattern of AGPs recognized by LM2 antibodies was slightly different from that found with LM6. LM6 labeling was homogenously localized in walls of all cells of proembryogenic masses (Figure 9A), while LM2 labeling appeared more intense for some cells of proembryogenic masses (Figure 9D). Somatic embryos at different developmental stages— the heart, torpedo (Figures 9B,E) and cotyledonary (Figures 9C,F) stages—showed intense LM6 and LM2 fluorescence labeling in their cell walls. In some cases, immunofluorescence signal of LM6 and LM2 highlighted not only cell walls but also certain areas of cytoplasm (Figures 9A,C–F), although cytoplasmic labeling was much more evident in the case of LM2. Specifically, LM2 labeling in the cytoplasm showed a localization pattern in small cytoplasmic spots (Figure 9F, inset), which would be compatible with structures of the secretory pathway, as previously reported for LM2 epitopes (Samaj et al., 2000; El-Tantawy et al., 2013).

### Effects of the Inhibition of Pectin Methylesterase Activity and the Blocking of AGPs on Somatic Embryogenesis

The results of the gene expression analyses of a *PME*, a putative *PMEI* and three AGPs, the PME enzymatic activity assay, and the dot blots and immunofluorescence assays indicated that pectin de-esterification and AGPs content increased in cell walls concomitantly with somatic embryogenesis progression. To analyze the possible involvement of pectin de-esterification and AGPs in the process of somatic embryogenesis, functional analyses with specific inhibitors were performed.

**FIGURE 10:**
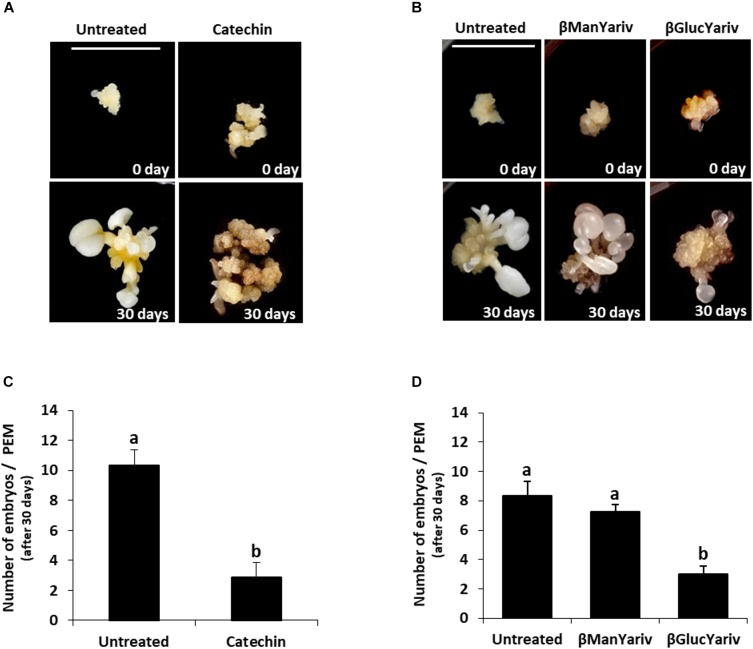
Effects of inhibition of PME activity by catechin and blocking of AGPs by Yariv reagents on somatic embryogenesis. **(A)** Catechin treatment. Left pictures: Untreated culture at the beginning of the treatment, 0 day, showing representative proembryogenic masses (PEMs) with a few small embryos arising from them, and after 30 days, when numerous embryos at different stages have developed. Right pictures: Catechin-treated culture at the beginning of the treatment showing similar PEMs than those of untreated cultures, and after 30 days, showing high proliferation of new PEMs but almost no differentiated embryos. **(B)** Yariv reagent treatment. From left to right: Untreated culture, β-mannosyl-treated culture, which does not bind AGPs, and β-glucosyl-treated culture, which binds AGPs. All cultures at the beginning of the treatment (0 day) show clusters of PMEs and a few small embryos. After 30 days of treatment, untreated and β-mannosyl-treated cultures show numerous and well developed embryos at different stages and various sizes, while β-glucosyl-treated culture shows PEMs that have grown very little and only a very few embryos. Bars represent 1 cm, for all pictures. **(C,D)** Quantification of the embryo production in untreated cultures and cultures treated with catechin **(C)** and Yariv reagents **(D)**. Columns represent mean values of the number of differentiated embryos per proembryogenic mass, after 30 days of treatment; bars represents the standard error of the mean (SEM). Different letters on columns indicate significant differences according to ANOVA and Tukey’s tests at *p* < 0.05.

Somatic embryogenesis cultures were treated with catechin PP60, which is a known inhibitor of PME activity (Lewis et al., 2008). Catechins from green tea extracts (called Polyphenon 60 or PP60) have been reported to inhibit PME activity *in vitro* in a wide range of plant species (Lewis et al., 2008). As small molecules with long shelf life and stability, they have been proposed as efficient inhibitors of PME by exogenous application in tissues (Lewis et al., 2008). Proembryogenic masses, formed after induction and multiplication, were selected and transferred to either control medium or medium containing 1.5 mg/ml catechin (Figure 10A and Supplementary Figures 2A,C). The development of treated cultures was evaluated and compared with control cultures. After 30 days, control cultures produced new proembryogenic masses and embryos at various stages of development, from globular to heart, torpedo and cotyledonary stages, as well as some mature embryos (Figure 10A and Supplementary Figure 2B). After 30 days, catechin-treated cultures showed numerous new proembryogenic masses that were produced by proliferation of the initial masses, and some small embryos at early globular stage; however, no further development and differentiation of embryos was observed (Figure 10A and Supplementary Figure 2D). Transfer of these embryogenic masses to culture medium without catechin led to recovery of the development and formation of numerous embryos, which were visible after 20 days in control medium (data not shown). These results indicated that inhibition of PME activity and, therefore, reduction of pectin de-esterification did not affect the initial stages of somatic embryogenesis. It even had the capacity to increase/promote the formation of proembryogenic masses, but it greatly impaired subsequent embryo differentiation and maturation steps.

To analyze the involvement of endogenous AGPs in somatic embryogenesis, functional analyses were performed by treatments with Yariv reagents that blocked AGPs. Two reagents were added to the culture media, β-Gluc-Yariv, which is known to aggregate AGPs, and β-Man-Yariv, which does not (Tang et al., 2006; Paulsen et al., 2014). Untreated cultures and β-Man-Yariv-treated cultures were used as controls. After 30 days, untreated cultures and cultures treated with β-Man-Yariv developed in a similar manner and showed similar somatic embryogenesis progression; they showed abundant new proembryogenic masses and the formation of numerous embryos at different developmental stages (Figure 10B and Supplementary Figures 3A–D). However, proembryogenic masses cultured in β-Gluc-Yariv-containing medium showed a much reduced development after 30 days, they produced some new proembryogenic masses and few embryos (Figure 10B and Supplementary Figures 3E,F), much less than in control and β-Man-Yariv-treated cultures (Figure 10, compare Supplementary Figure 3F with Supplementary Figures 3B,D), indicating that the precipitation of AGPs inhibited somatic embryogenesis, from the initial stages. The inhibition of embryogenesis progression produced by the blocking of AGPs by β-Gluc-Yariv was reversible; embryo development was recovered by transferring β-Gluc-treated samples to control medium. After around 20 days, recovered cultures showed larger and more developed embryos (Supplementary Figures 3G,H).

To quantitatively assess the effects of the treatments with catechin and Yariv reagents on somatic embryogenesis, the number of cotyledonary embryos developed after 30 days was quantified in untreated and treated cultures. The results showed that catechin treatment severely reduced the number of cotyledonary embryos formed in comparison with control cultures (Figure 10C). Regarding AGPs, treatment with β-Man-Yariv reagent did not result in significant differences in the number of cotyledonary embryos produced with respect to untreated cultures, while the number of embryos formed in β-Gluc-Yariv treated cultures was significantly lower (less than half) than the number formed in untreated cultures (Figure 10D). These results indicated that the inhibition of PME activity by catechin and the precipitation of endogenous AGPs by β-Gluc-Yariv reagent negatively affected the progression of somatic embryogenesis, suggesting a role for PME and AGPs during the process.

## Discussion

Increasing evidence indicates that growth and differentiation requires controlled remodeling of cell wall polysaccharide networks, resulting in changes in their mechanical properties to allow cell division and expansion to proceed normally (Barnes and Anderson, 2018). Cell wall components such as pectins and AGPs play crucial roles during organogenesis, as well as in somatic and zygotic embryogenesis (van Hengel et al., 2001, 2002; Baluska et al., 2002, 2005; Samaj et al., 2005; Seifert and Roberts, 2007; Geshi et al., 2013; Smertenko and Bozhkov, 2014). In the present study, we have analyzed whether pectins and AGPs could have a role in somatic embryogenesis of cork oak. The results obtained have revealed changes in PME activity and in the spatio-temporal patterns of distribution of esterified and de-esterified pectins during somatic embryogenesis, as well as their correlation with the expression pattern of a gene encoding a PME (*QsPME*). The results also showed changes in total AGP content, in the expression of several AGP genes (*QsLys-rich-AGP17, QsLys-rich-AGP18* and *QsAGP16L1)*, and in the distribution of certain AGP epitopes during somatic embryogenesis. The functional analyses with inhibitors of PME activity and Yariv reagents that precipitate AGPs indicated that both pectins and AGPs played a role in somatic embryogenesis of cork oak.

### Low PME Activity and Esterified Pectins Characterize Somatic Embryogenesis Initiation While Pectin De-Esterification Is Required for Embryo Differentiation

We have firstly investigated if somatic embryogenesis progression involved changes in pectin esterification levels, and their correlation with the gene expression of a PME and a putative PMEI, and the PME enzymatic activity, which would indicate the remodeling of the cell wall during somatic embryogenesis. PMEs are critical enzymes involved in cell wall remodeling during growth, and their activity is regulated by direct interaction with endogenous PMEIs. Large families of *PME* and *PMEI* genes have been identified in *A. thaliana*, and in other plant species; *PMEI* expression has been demonstrated in many of them, indicating that these protein inhibitors may be ubiquitously expressed in higher plants (Jolie et al., 2010). Two types of PMEs can be distinguished, depending on the presence or absence in their sequence of a pro region, similar to PMEI proteins (PMEI domain) preceding the active part (PME domain). Group 1/type II PMEs do not contain this pro region, while group 2/type I PMEs contain both PME and PMEI domains, but the PMEI is proteolitically released and eliminated before protein secretion to the cell wall (Pelloux et al., 2007; Wolf et al., 2009). However, much less information is available on PMEs and PMEIs of trees, particularly in *Q. suber*. Among the sequences annotated in the cork oak database^2^ (see footnote 1) there are some PMEs and very few PMEIs, with very little additional information available. For the expression analyses we have selected a PME gene sequence containing the PME catalytic domain, with high homology to sequences of other plant species, and named this sequence *QsPME.* Regarding the scarce PMEI sequences annotated in the cork oak database, we have selected a sequence that contained a PME Inhibitor domain but not a PME domain. Since this sequence was not a full length sequence, the possibility that the complete gene could include a PME domain, as in group 2/type I PMEs, cannot be completely ruled out. If this sequence corresponded to a PME gene, this would indicate the presence of a PME gene with a decreasing expression profile during somatic embryogenesis. Other possibility was to consider that the selected sequence (*QsPMEI)*, encoded a putative PME inhibitor. In any case, this small uncertainty about the real nature of the *QsPMEI* sequence does not affect the conclusions of the study, since the results clearly showed the increase in PME enzymatic activity and de-esterified pectins during somatic embryogenesis, together with the up-regulation of at least one PME gene (*QsPME*).

The expression profiles obtained for the two genes analyzed, *QsPME* and *QsPMEI*, showed that both genes were developmentally regulated during somatic embryogenesis. *QsPME* was expressed from early stages—in proembryogenic masses—and significantly increased its expression as embryogenesis proceeds, in heart, torpedo and cotyledonary embryos. *QsPMEI* only showed expression in proembryogenic masses while its expression was repressed at advanced stages of embryo differentiation. A recent report in another somatic embryogenesis system, *Brassica napus* microspore embryogenesis, has also demonstrated expression of a PME gene, *BnPME* (Solís et al., 2016). This report showed that *PME* transcript levels were very low at initial stages and increased with embryogenesis progression, in heart, torpedo and cotyledonary embryos, that is, an analogous pattern to that found in *Q. suber* somatic embryogenesis. Differential temporal and spatial expression of *PME* and *PMEI* genes has been proposed as a major mechanism to regulate the endogenous PME activity (Jolie et al., 2010). In cork oak, the PME enzymatic activity assay demonstrated a progressive increase in this activity throughout somatic embryogenesis, correlating with *QsPME* gene expression profile. If we consider that *QsPMEI* sequence encoded a real PMEI, we can hypothesized that during initiation of somatic embryogenesis, *PMEI* may inhibit PME activity and pectins are mostly be in a highly esterified state. In contrast, at subsequent somatic embryogenesis stages, *PME* expression was up-regulated and *PMEI* expression was repressed, permitting the activity of PMEs and the de-esterification of pectins, which accompany embryo differentiation. If *QsPMEI* sequence would be a group 2/type I PME, its expression profile would indicate that this gene was not relevant in the regulation of the esterification levels of pectins during somatic embryogenesis. Since PMEs and PMEIs belong to large multigene families, probably other genes could participate in the transcriptional regulation of PME activity during somatic embryogenesis in cork oak. Further work will be necessary to precisely identify all the genes (in the cork oak genome) and proteins responsible for the reported increase in PME activity.

In agreement with this, immunolocalization results have revealed that cell walls of proembryogenic masses exhibited a high signal of esterified pectins and very low or no signal of de-esterified pectins, indicating a predominant presence of highly esterified pectins at the initial stages of somatic embryogenesis. Previous studies have shown the presence of high proportions of esterified pectins as markers of proliferative cells, in root meristems and early microspore proembryos, in *Capsicum annum* and *B. napus* (Bárány et al., 2010b,a; Solís et al., 2016), as well as in early microspore embryos of *Q. suber* (Rodriguez-Sanz et al., 2014). Proembryogenic masses are characterized by their capacities to proliferate and to initiate embryo formation. Our results in proembryogenic masses of cork oak indicated that early proliferative stages of somatic embryogenesis initiation were associated with low PME activity and high levels of esterified pectins.

At advanced stages of somatic embryogenesis, in differentiating embryos, signal for both types of pectins, highly and low-esterified pectins, were observed in most embryo cells, with different intensities that suggested variable proportions and levels of de-esterification in cell walls during embryo development. Globally, as shown by immuno dot blot and immunofluorescence assays, differentiating embryos exhibited slightly higher signals for de-esterified pectins than for esterified ones, indicating that de-esterification of pectins may accompany embryo development progression. In cork oak, the presence of esterified pectins revealed via immunolocalization with JIM7 antibodies has also been reported in all stages of pollen development (Costa et al., 2015) and unfertilized female tissues (Lopes et al., 2016). Interestingly, the differentiating epidermis of heart, torpedo and cotyledonary somatic embryos showed a much higher proportion of de-esterified pectins and almost no presence of esterified pectins. Decreasing methylesterification levels have been found from proliferating to differentiating tissues in various plant species (Jolie et al., 2010). Specifically in embryogenesis, several PMEs are expressed during silique development in *Arabidopsis* (Louvet et al., 2006), and during microspore and zygotic embryogenesis progression in *B. napus* (Solís et al., 2016). In these reports, the increasing activity of PME and pectin de-esterification levels have been reported as crucial factors in the change of cell wall properties for embryo differentiation (Solís et al., 2016). Our results in *Q. suber* showed similar dynamics of PME activity and pectin de-esterification status during somatic embryogenesis progression, which may indicate that the expression of PME and PME inhibitors may contribute to the temporal regulation of biomechanical properties of cell walls through the balance between highly and low-esterified pectins.

In our study, we have used treatments with catechin (PP60), which inhibits PME activity, to analyze the role of PME in somatic embryogenesis of *Q. suber*. Consistent with a role for pectin de-methylesterification in somatic embryogenesis progression, the inhibition of PME activity by pharmacological treatments with catechin PP60 resulted in the impairment of embryo differentiation. However, catechin treatment did not affect the proliferation of proembryogenic masses and embryogenesis initiation. During organogenesis initiation in *Arabidopsis*, auxin regulates the cell wall stiffness that requires de-methyl-esterification of pectins (Peaucelle et al., 2011; Braybrook and Peaucelle, 2013), a process that is precisely controlled by the balance of activity between PME enzymes and PME inhibitors (Jolie et al., 2010). The results of the PME inhibition by catechin treatment—together with the results regarding *PME* and *PMEI* expression, PME activity, immuno dot blot and localization of esterified and de-esterified pectins—support the idea that PME activity and its endogenous regulator PMEI are involved in the process of somatic embryogenesis and that the PME-mediated configuration of pectins could be a crucial factor for somatic embryo differentiation in *Q. suber*. Taken together, these results also indicate that pectin de-esterification is required for somatic embryo differentiation in cork oak.

### AGP Level of Accumulation Progressively Increases and Is Required in Somatic Embryogenesis

Increasing evidence has shown that AGPs have a role in reproductive tissues and in embryo development (Zhong et al., 2011; Geshi et al., 2013). In *Arabidopsis*, 85 AGPs have been identified (Showalter et al., 2010), however, there is little information on AGP genes in non-model species due to the heterogeneous and complex structure of these macromolecules (Pereira et al., 2016; Han et al., 2017). The analyses reported here have revealed the up-regulation of three different AGP genes, *QsLys-rich-AGP17*, *QsLys-rich-AGP18*, and *QsAGP16L1*, and a progressive increase in AGP level of accumulation, by precipitation with β-Gluc-Yariv, from early to advanced stages of somatic embryogenesis. Furthermore, these findings correlated with temporal profiles of certain AGP epitopes, as revealed by immuno dot blot and immunofluorescence assays.

Arabinogalactan proteins belong to large multigene families which have not been fully identified in many plant species. In particular, there is very scarce information about AGP sequences in the cork oak database. Among them, we have selected three sequences of two types of AGPs—two Lys-rich-AGPs and one AG peptide. Recent reports regarding *Brassica rapa* and *Arabidopsis*, about homologous genes of the selected cork oak AGP sequences, have related them with embryo formation, which suggested that they could be also expressed in somatic embryogenesis. Expression of *AGP17*, *AGP18*, and *AGP16* has been reported in siliques containing embryos (Han et al., 2017); furthermore, *AGP18* has been described as essential in female gametophyte formation (Acosta-García and Vielle-Calzada, 2004). *QsAGP16L1* (*QsAGP16-like1*) has been recently identified (Costa et al., 2015) in the cork oak database as an AG peptide with homology with the *AtAGP16* of *Arabidopsis*, with expression during pollen development in the two species (Nguema-Ona et al., 2012; Costa et al., 2015). The expression of Lys-rich *AGP18* gene, has been detected in *Arabidopsis* female reproductive tissues (Acosta-García and Vielle-Calzada, 2004), roots, flowers, and stems (Yang and Showalter, 2007; Yang et al., 2007), as well as in vegetative and reproductive development in pepper (Verdugo-Perales et al., 2018). However, information about the expression of AGP genes in somatic embryogenesis is very scarce. Differential expression of *BnAGP-Sta39-4* was reported at early stages of microspore embryogenesis in *B. napus* (El-Tantawy et al., 2013). Our results have demonstrated the up-regulation of three different AGP genes, *QsLys-rich-AGP17, QsLys-rich-AGP18* and *QsAGP16L1*, during somatic embryogenesis, suggesting a role for AGPs in somatic embryo development in this woody species.

Our study has shown the increase in transcript accumulation for three AGP-encoding genes during somatic embryogenesis. Moreover, we have demonstrated the progressive overall AGP accumulation, by precipitation with β-Gluc-Yariv reagent. The levels of various glycan AGP epitopes also increased during somatic embryogenesis of cork oak, as revealed by immuno dot blot and immunofluorescence assays. Immunofluorescence results showed the localization of AGPs mostly in cell walls of proembryogenic masses and somatic embryos. Some AGP epitopes (mostly those recognized by LM2 antibodies) showed a distribution pattern in cell walls and small cytoplasmic spots. Previous reports have shown localization of AGP antigens at early stages of microspore embryogenesis of *B. napus* (El-Tantawy et al., 2013), with the pattern of distribution of LM2 antigen being analogous to that observed in cork oak somatic embryo cells, with localization in cell walls and small cytoplasmic spots that were proposed as secretion subcompartments (El-Tantawy et al., 2013). In rice and carrot suspension cells, LM2 antigen was present in AGPs secreted into the medium (Smallwood et al., 1996). Moreover, LM2-AGP antigen has been associated with subcellular elements of the secretory pathway in plant cells that secrete AGPs (Samaj et al., 2000). Furthermore, a recent report has shown that somatic embryogenesis cultures of cotton produced and secreted AGPs and that when these AGPs were incorporated into culture medium, somatic embryogenesis was promoted (Poon et al., 2012). The localization patterns of AGPs, revealed by the immunofluorescence assays in our study, are consistent with their localization in cell walls and we hypothesize their possible secretion during cork oak somatic embryogenesis, as is reported in other *in vitro* embryogenic systems. Although we cannot rule out the possibility that other AGPs could have different expression patterns in somatic embryogenesis, the results indicate that globally there was a large proportion of AGPs that increased their levels during somatic embryo development in cork oak, suggesting their involvement in this process.

The results of the β-glucosyl Yariv treatments have revealed that the inactivation of AGPs impaired somatic embryogenesis in *Q. suber*, supporting the idea of an active role of these macromolecules in the process. The blocking of AGPs by Yariv reagent reduced embryogenesis initiation rates and inhibited embryogenesis progression, which indicates that AGPs were involved in the development of embryos and were required for the initiation and progression of somatic embryogenesis of cork oak. The treatment with Yariv reagents has been used since these reagents are reliable cytochemical compounds for exploring AGP functions (Seifert and Roberts, 2007; Jolie et al., 2010). By using Yariv reagents to inactivate AGPs in established *in vitro* embryogenesis cultures, a role for AGPs has been proposed in the initiation and maintenance of microspore embryogenesis in *B. napus* (Tang et al., 2006) and *in vitro* zygotic embryogenesis of tobacco (Yu and Zhao, 2012). The results of the present study have demonstrated—in a woody species—that AGPs play a key role in somatic embryogenesis, from early stages.

### Modifications in Pectins and AGPs Suggest Cell Wall Remodeling During Somatic Embryogenesis

In the present study, we have shown changes in pectins and AGPs that were associated with somatic embryogenesis initiation and progression; specifically pectin de-esterification and AGP levels increased throughout the process. Moreover, specific inhibitors of PME activity and reagents that block AGPs impaired somatic embryogenesis, revealing that both de-esterified pectins and a large group of AGPs were involved in the process and were required for embryogenesis progression. The localization of these macromolecules in the cell wall of proembryogenic masses and developing somatic embryos has also been shown, suggesting that during somatic embryogenesis the cell wall is remodeled, with modifications of two of their main components, pectins and AGPs.

Increasing evidence supports the idea that growth and differentiation requires controlled remodeling of wall polysaccharide networks, structure and components, although little is known about the processes that regulate the cell wall remodeling (Barnes and Anderson, 2018; Voiniciuc et al., 2018). Dynamics of pectin esterification levels, regulated by PMEs and PMEIs, are thought to be involved in proliferation and differentiation events of numerous developmental processes, including embryogenesis (Bárány et al., 2010a; Rodriguez-Sanz et al., 2014; Solís et al., 2016; Corredoira et al., 2017). During these processes, pectin de-esterification can act by modulating the mechanical properties of the wall, such as its stiffness, charge or susceptibility to degradation (Barnes and Anderson, 2018). AGPs have also been proposed as modulators of cell wall mechanics (Seifert and Roberts, 2007). The specific and stable binding of AGPs to β-glucosyl-Yariv reagent suggests that endogenous AGPs can be trapped by pectins through an analogous interaction that might occur in the cell wall between AGPs and the β-galacturonan domain of pectins—an association that has already been observed in various systems and may modulate the mechanical properties of the pectic matrix (Seifert and Roberts, 2007; Liao et al., 2011). In addition, a possible further mechanical role of AGPs in stiffening the cell wall by oxidative crosslinking has been proposed (Seifert and Roberts, 2007). The results of the present study have shown that the degree of pectin de-esterification and AGP levels of accumulation both increased during somatic embryogenesis and are required for its progression, in relation to proliferation and differentiation events, which would promote the cell wall remodeling during the process. The new findings also provide new insights into the regulating mechanisms of somatic embryogenesis for potential applications in improving somatic embryogenesis yield in tree breeding programs.

## Author Contributions

YP-P and EC performed most of the experimental work. YP-P carried out microscopy analyses, immunofluorescence, immuno dot blot assays, Yariv dot blot and pharmacological *in vitro* treatments, contributed to gene sequence searching and qPCR assays, and prepared the figures. EC performed most *in vitro* cultures, the search and selection of gene sequences, designed primers, performed all qPCR assays, and contributed to immuno dot blots. EB performed the PME activity assays. M-TS performed some immunocytochemical assays. IB performed some *in vitro* cultures. AG-G and BP selected and collected material from trees in the field, and generated somatic embryogenesis cultures. MR participated in the discussion of the results. PT conceived, designed, and supervised the experimental work, analyzed the results, elaborated the conclusions, and wrote the manuscript. All authors read and approved the final manuscript.

## Conflict of Interest Statement

The authors declare that the research was conducted in the absence of any commercial or financial relationships that could be construed as a potential conflict of interest.

## Footnotes

1 http://www.corkoakdb.org/
