# Supplementary material for: Pectin De-methylesterification and AGP Increase Promote Cell Wall Remodeling and Are Required During Somatic Embryogenesis of Quercus suber
Source: Front Plant Sci. 2019 Jan 8;9:1915. doi: 10.3389/fpls.2018.01915 (PMC6331538; doi:10.3389/fpls.2018.01915)
Supplement: Supplementary file 1 [file Data_Sheet_1.PDF]

Supplementary Material

Pectin de-methylesterification and AGP increase promote cell wall remodeling and are required during somatic embryogenesis of *Quercus suber*

Yolanda PÉREZ-PÉREZ[1,a], Elena CARNEROS[1,a], Eduardo BERENGUER[1], María-Teresa SOLÍS[1,2], Ivett BÁRÁNY[1], Beatriz PINTOS[2], Aránzazu GÓMEZ-GARAY[2], María C. RISUEÑO[1], Pilar S. TESTILLANO[1,*]

[1]Pollen Biotechnology of Crop Plants group, Biological Research Center, CIB-CSIC, Madrid, Spain
[2]Dept. Genetics, Microbiology and Physiology, Univ. Complutense of Madrid, Madrid, Spain

[a]Both authors contributed equally

[*]Corresponding author:
Dr. Pilar S. Testillano
testillano@cib.csic.es

Supplementary Tables

Supplementary Table 1: List of antibodies used in the study.

Antibody	Target	Dilution for immunofluorescence (IF) and immuno dot blot assays (DB)	Reference
JIM5	Low/De-esterified pectins	IF: undiluted<br>DB: 1/100	Knox *et al.* 1990
JIM7	Highly-esterified pectins	IF: 1/5<br>BD: 1/100	Knox *et al.* 1990
LM19	De-esterified pectins	IF: 1/5<br>DB: 1/100	Vehertbruggen *et al.* 2009
LM20	Highly-esterified pectins	IF: 1/5<br>DB: 1/100	Vehertbruggen *et al.* 2009
LM2	β-linked-GlcA in AGP glycans	IF: 1/20<br>DB: 1/200	Smallwood *et al.* 1996
LM6	α-(1-5)-L-arabinan in AGP glycan, and arabinan of RGI domain of pectins)	IF: 1/5<br>DB: 1/100	Willats *et al.* 1998
JIM14	AGP glycan	IF: 1/5<br>DB: 1/100	Knox *et al.* 1991, Yates *et al.* 1996

Supplementary Table 2: List of primers used for qRT-PCR

Gene abbreviation	Accession nº	Primer sequences 5'3' (F/R)	Product size (bp)
QsPME	QS073834.0*	AGTTGCAGAAGCGTATGTCC / GCAGGAGTTTACACGGAGAA	156
QsPMEI	QS125571.0*	ACTCCACCATAGTCACCAGC / GCCAAAACCACTGTCGATGC	170
QsAGP16L1	QS001499.0*	AGTGGCTCAGGCACAATCTC / CTACGGTGATGATGAGGCGT	157
QsLys-rich-AGP18	QS117616.0*	TGGTGCTTGTGGTTTAGACG / TGGCATTCGCATTGATCTGC	137
QsLys-rich-AGP17	QS005141.0*	GACAGTGGGGTGGAGAAGAT / ACCAACAATAGGAGAGCCCA	80
QsACT	EU697020.1**	CCTGATGGGCAGGTTATCACA / GCTTCAATGAGAGATGGCTGG	80

* CorkOakDB (www.corkoakdb.org); **GenBank (www.ncbi.nlm.nih.gov/genbank)

Supplementary Figure 1

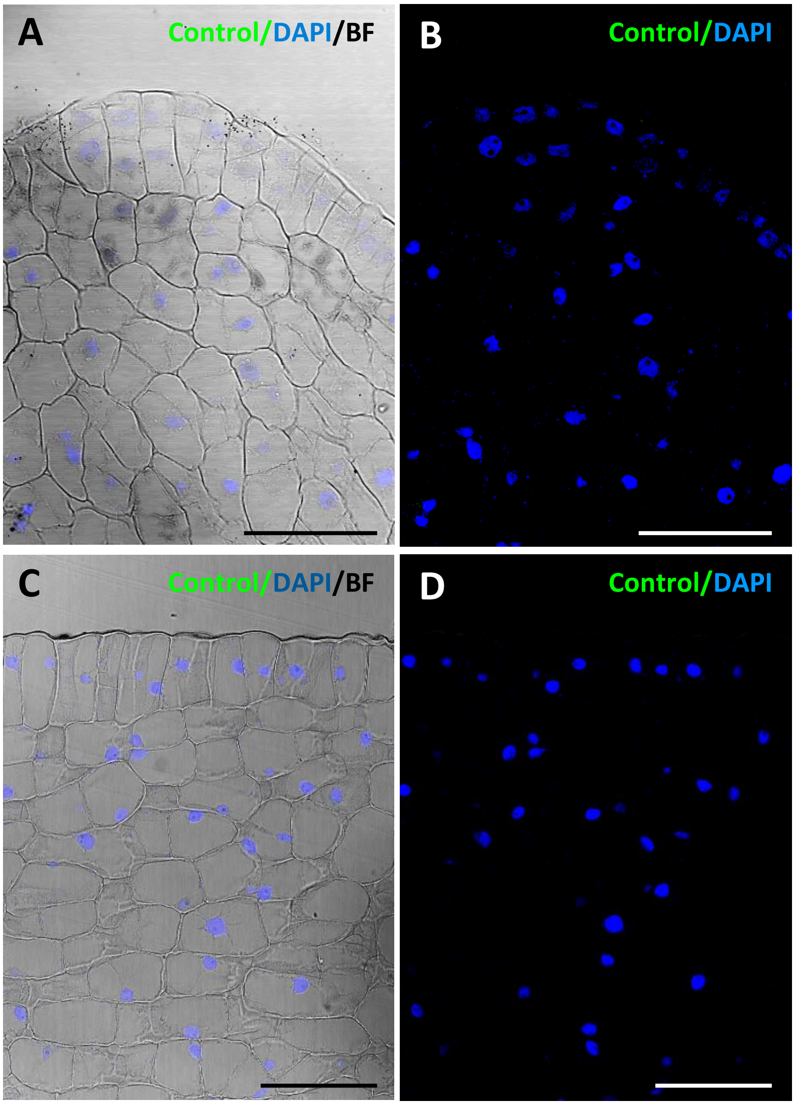

Supplementary Figure 1: **Negative controls of immunofluorescence experiments by avoiding the primary antibody.** The same embryo regions are visualized under different microscopy modes. **A, B**: Torpedo embryo. **C, D**: Cotyledonary embryo. A, C: Merged micrographs of bright field (BF), DAPI staining (blue signal) for nuclei, and secondary antibody (green signal). B, D: Confocal merged images of DAPI staining (blue signal) for nuclei, and secondary antibody (green signal). Bars represent 50 µm.

Supplementary Figure 2

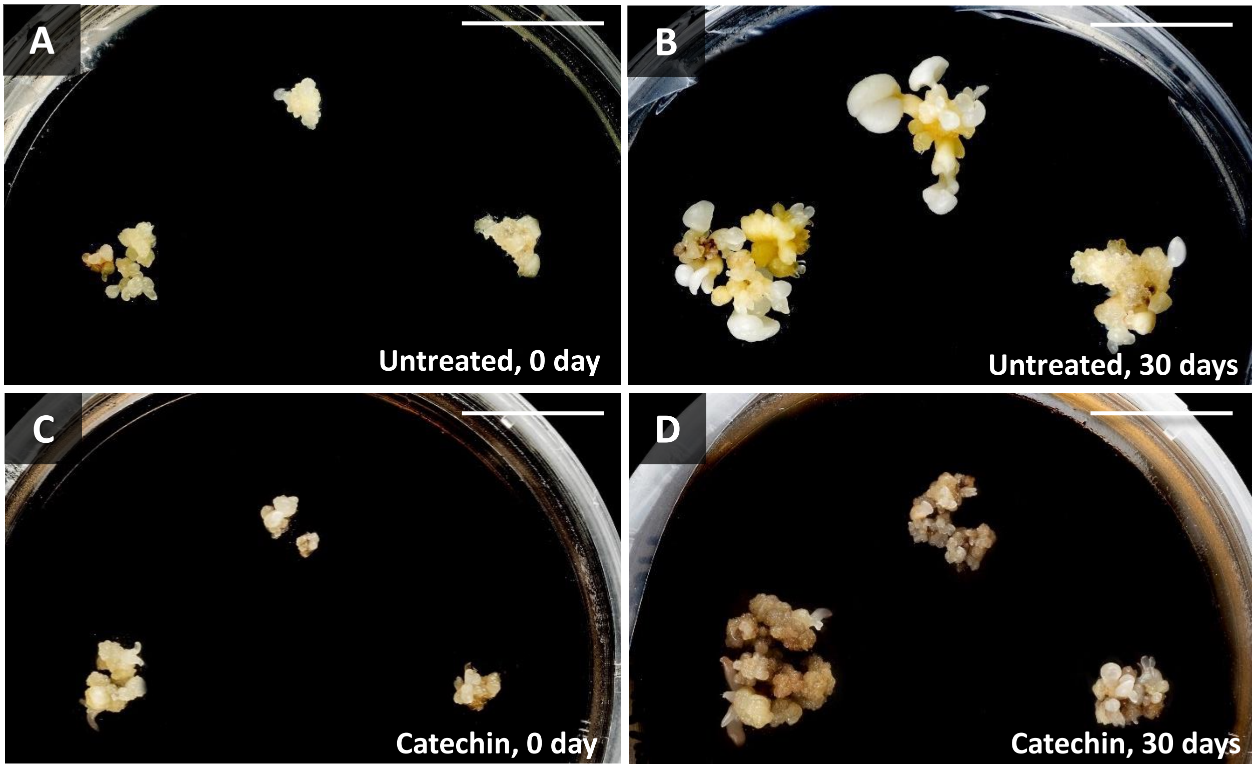

Supplementary Figure 2: Effects of inhibition of PME activity by catechin on somatic embryogenesis. A, B: Untreated culture at the begining of the treatment, day 0 (A), showing proembryogenic masses with a few small embryos arising from them, and after 30 days (B), when numerous embryos at different stages have developed**. C, D:** Catechin-treated culture at the begining of the treatment (C), showing similar proembryogenic masses than those of untreated cultures, and after 30 days (D), showing high proliferation of new proembryogenic masses but almost no differentiated embryos. Bars represent 1 cm.

Supplementary Figure 3

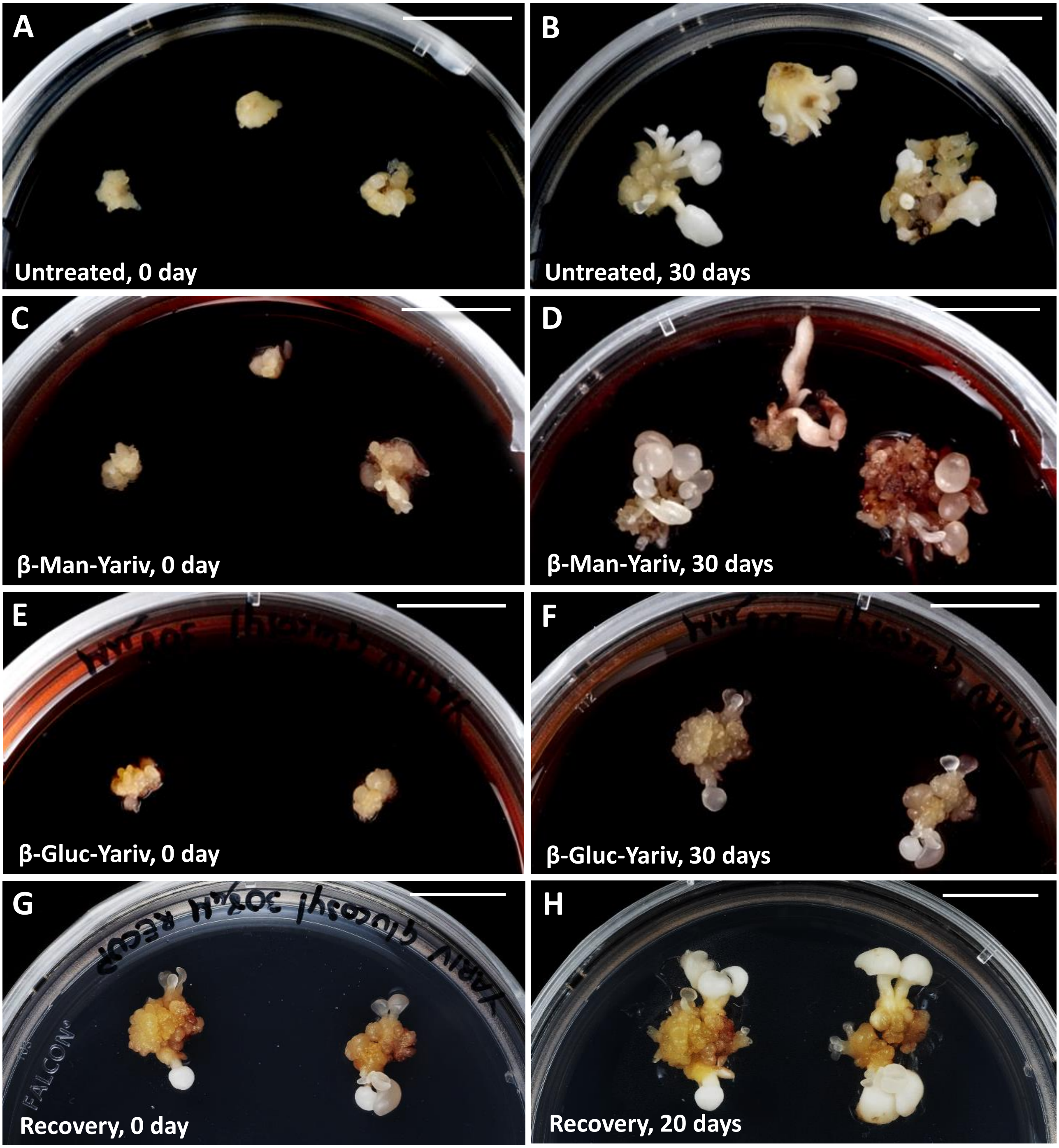

Supplementary Figure 3: Effects of blocking of AGPs by Yariv reagents on somatic embryogenesis. A, B: Untreated culture**. C, D:** β-Mannosyl-treated culture, which does not bind AGPs. **E, F:** β-Glucosyl-treated culture, which binds AGPs. A, C, E: Cultures at the beginning of the treatment with clusters of proembryogenic masses and a few small embryos. B, D, F: Cultures after 30 days of treatment. Untreated (B) and β-Mannosyl-treated (D) cultures show numerous and well developed embryos at different stages and various sizes. β-Glucosyl-treated culture (E) show proembryogenic masses that have grown very little and a only a few embryos. **G, H:** Recovery of cultures after 30 days of β-Glucosyl-treatment followed by transfer to control medium (without β-Glucosyl-Yariv). G: Same culture samples than in E, at the beginning of the recovery (day 0 in control medium). H: Recovery culture after 20 days in control medium; embryos have developed and exhibit larger size, more prominent cotyledons and an opaque colour. Bars represent 1 cm.
